# *Inonotus obliquus* Crude Melanin Ameliorates DSS-Induced Colitis with Modulation of Gut Microbiota and Neutrophil pro-NETotic Activation

**DOI:** 10.3390/nu18111733

**Published:** 2026-05-28

**Authors:** Hongxia Yuan, Yajie Liu, Xinyi Wang, Zhijun Yang, Chunmei Wu, Fan Yang, Qingshan Li

**Affiliations:** 1 Shanxi Key Laboratory of Innovative Drug for the Treatment of Serious Diseases Basing on the Chronic Inflammation, College of Traditional Chinese Medicine and Food Engineering, Shanxi University of Chinese Medicine, Jinzhong 030619, China; yuanhongxia609@sxtcm.edu.cn (H.Y.); liuyajie@sxtcm.edu.cn (Y.L.); wangxinyi1@sxtcm.edu.cn (X.W.); yangzhijun@sxtcm.edu.cn (Z.Y.); wuchunmei@sxtcm.edu.cn (C.W.); 2 Medicinal Basic Research Innovation Center of Chronic Kidney Disease, Ministry of Education, Shanxi Medical University, Jinzhong 030619, China; fanfan42@sxmu.edu.cn

**Keywords:** *Inonotus obliquus* crude melanin, ulcerative colitis, gut microbiota, neutrophil pro-NETotic activation, intestinal mucosal barrier

## Abstract

**Background**: *Inonotus obliquus*, a fungus known for its edible and medicinal properties, has traditionally been used as a herbal tea to relieve gastrointestinal disorders. Melanin, a major active constituent of this fungus, exhibits antioxidant, anti-inflammatory, and immune-modulating effects. This study aimed to investigate the therapeutic potential and mechanisms of *Inonotus obliquus* crude melanin (IOM) in treating dextran sulfate sodium (DSS)-induced colitis in mice. **Methods**: The study assessed colonic inflammation, mucosal damage, and intestinal barrier integrity. It also measured the levels of proinflammatory cytokines and oxidative stress markers. Gut microbiota composition was analyzed using 16S rRNA gene sequencing following IOM treatment. Additionally, label-free quantitative proteomic analysis was performed to explore the underlying mechanisms. **Results**: IOM administration significantly ameliorated colitis symptoms, strengthened the intestinal barrier, and reduced inflammation and oxidative stress in a dose-dependent manner. Furthermore, IOM modulated gut microbiota composition by increasing the relative abundance of *Lactobacillus* and *Muribaculaceae*, while reducing that of *Bacteroides*, *Escherichia-Shigella*, and *Romboutsia*. Proteomic analyses revealed that IOM treatment regulated the neutrophil pro-NETotic signaling pathway, which was further verified by immunohistochemistry or Western blot analysis of key pro-NETotic markers (e.g., PAD4, CitH3, MPO). Importantly, the relative abundances of these bacterial taxa were significantly correlated with the ulcerative colitis (UC) progression and neutrophil pro-NETotic activation. **Conclusions**: IOM mitigates DSS-induced colitis alongside the modulation of gut microbiota and the suppression of neutrophil pro-NETotic activation, suggesting its potential as a functional food ingredient for the management of UC.

## 1. Introduction

Ulcerative colitis (UC) is an idiopathic recurrent intestinal inflammatory disease featured by sustained mucosal lesions that arise from the rectum and gradually extend to the proximal colon [1]. Clinically, UC manifests as diarrhea, abdominal pain, hematochezia, and weight loss, which seriously affect daily life and bring heavy social and economic costs [2,3]. Despite the widespread clinical application of conventional therapeutics including aminosalicylic acids, corticosteroids and thiopurines, these regimens are severely limited by undesirable adverse reactions and dismal long-term remission rates, thus creating an urgent imperative for the development of safer, more effective and sustainable alternative interventions [4,5].

*Inonotus obliquus* (Ach. ex Pers.) Pilát, a member of the family *Hymenochaetaceae* and commonly known as Chaga, is an edible and medicinal fungus predominantly located in the cold regions of Eurasia and northeastern China, where it primarily colonizes elm, birch, and Alnus cremastogyne trees [6,7]. Traditionally consumed as a health-promoting beverage, Chaga exhibits notable antioxidant and anti-inflammatory properties and demonstrates promising potential in the amelioration of various gastrointestinal disorders [8,9]. Oral administration of *Inonotus obliquus* extract has been shown to mitigate acute inflammation in dextran sulfate sodium (DSS)-induced colitis in murine models [10]. *Inonotus obliquus* is known to contain a diverse array of bioactive constituents, including polysaccharides, triterpenoids, polyphenols, and melanin, among others [11,12,13]. While the polysaccharides derived from *Inonotus obliquus* have been reported to exert therapeutic effects on colitis [12], the therapeutic potential of melanin, another abundant constituent in its extracts, remains inadequately understood [13].

Accumulating evidence indicates that the pathogenesis of UC involves excessive oxidative stress, persistent inflammation, and dysregulated immune responses [14,15]. Melanin from *Inonotus obliquus* has been shown to possess potent antioxidant, anti-inflammatory, and immunomodulatory activities [13,16]. Based on these findings, we propose that dietary supplementation with *Inonotus obliquus* crude melanin (IOM) may serve as an effective strategy for alleviating UC. However, as a fungal-derived melanin, IOM typically exhibits poor solubility and a macromolecular polymeric structure, which hinders its absorption in the body under natural conditions [13]. Importantly, many bioactive compounds with low oral bioavailability exert their biological effects primarily through modulation of the gut microbiota rather than through systemic absorption [17,18]. These compounds can serve as substrates for beneficial gut bacteria, enhance their proliferation, inhibit the growth of pathogenic bacteria, and subsequently regulate intestinal metabolic homeostasis to achieve therapeutic outcomes [19,20].

Moreover, gut microbial dysbiosis has been recognized as a key driver in the onset and progression of UC [21,22]. Typical alterations include decreased microbial diversity, depletion of beneficial bacteria such as *Lactobacillus* and *Muribaculaceae*, and overgrowth of opportunistic pathogens, including *Escherichia-Shigella*, *Bacteroides*, and *Romboutsia* [23,24,25]. These disturbances further disrupt intestinal barrier function, trigger excessive inflammatory responses, oxidative stress, and exacerbate mucosal injury [26,27,28,29]. Therefore, interventions targeting the restoration of gut microbial homeostasis hold great promise for alleviating colonic inflammation and managing UC.

In this study, we investigated the therapeutic effect of IOM in DSS-induced UC mice. Furthermore, the underlying mechanism was elucidated using 16S rRNA gene sequencing and label-free proteomics. This research is anticipated to promote the development and application of IOM as a natural bioactive component in functional foods and pharmaceuticals for the management of UC.

## 2. Materials and Methods

### 2.1. Materials

*Inonotus obliquus* was provided by Daxinganling Senyuanshan Products Co., Ltd. (Daxinganlian, China). Dextran sulfate sodium (DSS) was acquired from Dalian Meilun Biotech Co., Ltd. (Dalian, China). Mesalazine (batch no. 210608) was from Sunflower Group Jiamusi Luling Pharmaceutical Co., Ltd. (Jiamusi, China). The enzyme-linked immunosorbent assay (ELISA) kits for TNF-α, IL-6 and IL-1β were purchased from Shanghai Fankel Industrial Co., Ltd. (Shanghai, China). Assay kits for SOD, MDA and MPO were purchased from Jiancheng Institute of Bioengineering, Nanjing. Antibodies against peptidylarginine deiminase 4 (PAD4) and citrullinated histone H3 (CitH3) were from Proteintech and Abcam, respectively. Antibodies against zonula occludens-1 (ZO-1), Occludin, Claudin-1, mucin 2 (MUC2), MPO, lymphocyte antigen 6 complex locus G (Ly6G) and β-actin were from Servicebio. All other chemicals employed in the experiment met the analytical grade standard.

### 2.2. Extraction of IOM

The IOM was prepared by modifying an existing method [13]. The *Inonotus obliquus* specimens utilized in this investigation displayed morphological features such as spherical, oblate, or irregular masses of varying sizes, with diameters ranging from 25 to 40 cm. These specimens were sessile and exhibited verrucose protrusions on their surfaces. The outer surface was characterized by a grayish-black color and an irregular, deeply furrowed, and wrinkled texture. The fungus possessed a hard consistency, with a thin outer layer approximately 5 mm thick, which was dark brown, while the internal flesh varied from yellow to yellowish-brown. It emitted a subtle odor and had a mildly sweet taste. *Inonotus obliquus* powder was first extracted with water at a 1:10 ratio for three hours, followed by three one-hour extractions. The combined extracts were concentrated, mixed with ethanol to 75%, and incubated at 4 °C overnight. The pellet was harvested, lyophilized, acidified to pH 2 with HCl, and centrifuged again. After washing with dilute HCl, neutralizing with NaOH, dialyzing, and freeze-drying, the melanin extract was obtained. This was hydrolyzed with concentrated HCl at 100 °C for 24 h, centrifuged, washed to neutral pH, and freeze-dried to yield purified IOM. The yield of IOM was determined to be 7.98% ± 0.37%.

### 2.3. Physicochemical Characterization of IOM

The IOM powder was gold-sprayed for 50 s to enhance conductivity and attached to a conductive adhesive for testing. Its surface was examined with an SU8600 Scanning Electron Microscope (SEM). The sample was reconstituted in 0.1 mol/L NaOH to 0.05 mg/mL, and its UV-visible absorption spectrum was detected from 200 to 700 nm with a UV-visible spectrophotometer (Ultra-3660), using NaOH as the blank control. Fourier transform infrared (FTIR) spectra of IOM were recorded using a Nicolet iS10 spectrometer. Samples mixed with KBr (1:100) were compressed into tablets, and spectra were captured from 4000 to 400 cm^−1^. For Electron Paramagnetic Resonance (EPR) spectra, a weighed IOM sample was placed in a quartz tube under a magnetic field at 25 °C using a Bruker A300, with specific conditions: 9.85 GHz frequency, 100 kHz modulation, 1 G amplitude, 19.71 mW microwave power, 61.44 s scan time, 3510.00 G central field, 150 G scan width, and 3480.00 G static field. Thermogravimetric analysis (TGA) using a Hitachi STA200 assessed the thermal stability of IOM by heating 10 mg samples at 10 °C per minute. The temperature was ramped from ambient to 1000 °C in a nitrogen atmosphere, and the maximum decomposition temperature (from DTG curves) and weight loss (from TG curves) were identified. The purity of the IOM was determined by High Performance Liquid Chromatography (HPLC) using a Waters e2695 system with a Diamonsil C18 column at a wavelength of 217 nm (see Supplementary Table S1). C, H, N, and S contents in melanin were measured using an Elementar Vario EL cube analyzer by burning samples and analyzing gaseous products.

### 2.4. Animal Model and Treatment

Male C57BL/6 mice, aged 6–8 weeks, were provided by SiPeiFu Biotechnology Co., Ltd., Beijing, China. All animal experiments were approved on 2 July 2023 by the Ethics Committee of Shanxi University of Chinese Medicine (approval code: 202307332) and adhered to ethical standards for animal care. All animals were housed in a controlled environment with temperature (23 ± 2 °C), humidity (30 ± 5%), and a regular 12 h light-dark cycle. Following one week of adaptation, the animals were divided into six experimental groups (*n* = 10 per group): Control, Model, IOM-L (50 mg·kg^−1^·d^−1^), IOM-M (100 mg·kg^−1^·d^−1^), IOM-H (200 mg·kg^−1^·d^−1^), and Mesalazine (5-ASA, 290 mg·kg^−1^·d^−1^).

Except for the Control group, all mice were given DSS (2.5%) in their drinking water for one week to establish the experimental model. Post-DSS treatment, mice were given daily oral doses of IOM or 5-ASA for 7 days. For the Control and Model groups, 0.5% CMC-Na solution was used as the vehicle control during the same period. 24 h after the final dose, all mice were humanely sacrificed. Colon tissues were isolated, measured, and divided for paraffin embedding and cryopreservation at −80 °C. Blood samples were collected for serum extraction, with serum stored at −80 °C for future experiments.

### 2.5. Disease Activity Index (DAI) Score Assessment

DAI scores were employed to assess UC severity, based on daily measurements of body weight, stool form, and stool occult blood. These scores specifically represented the arithmetic mean of the three aforementioned parameters. A comprehensive breakdown of the DAI scoring criteria is provided in Supplementary Table S2.

### 2.6. Histopathological Analysis

Following fixation, decolorization, and embedding, colon tissues were cut into 4-μm-thick sections. Colonic tissue sections were stained with hematoxylin and eosin (HE) for histopathological observation. Alcian Blue Periodic Acid Schiff (AB-PAS) staining was used to evaluate mucin in the colonic mucosa. Colonic tissue damage was evaluated according to Supplementary Table S3, and the goblet cell area was quantified using ImageJ software (v1.54p).

### 2.7. ELISA Analysis

Serum levels of TNF-α, IL-1β, and IL-6 were determined by ELISA, with all procedures performed in accordance with the manufacturer’s instructions.

### 2.8. Colon Tissue Oxidation Level Assay

Colon tissues were collected for the measurement of MDA, SOD, and MPO levels using corresponding detection kits, in strict accordance with the manufacturer’s protocols.

### 2.9. Immunohistochemical Staining and Scoring

Immunohistochemistry (IHC) was used to evaluate the expression of ZO-1, Occludin, MUC2, Claudin-1, MPO, and Ly6g in colon tissues, following established experimental methods [30]. After dewaxing and hydration, paraffin sections were immersed in an antigen repair solution. After inactivation of endogenous peroxidase with 3% H_2_O_2_, the sections were blocked with 3% Bovine Serum Albumin (BSA) for 30 min at room temperature to prevent non-specific binding. Overnight incubation with primary antibodies (1:500, Servicebio) was performed, followed by 50 min of incubation with secondary antibodies. The reaction was stopped with water after diaminobenzidine (DAB) staining, and subsequently, the DAB-positive regions were observed under a microscope.

### 2.10. Quantitative Real-Time PCR

Total RNA was isolated from frozen colonic tissues using TRIzol reagent. First-strand cDNA was synthesized using a Bio-Rad C1000 Touch Thermal Cycler (1000 Alfred Nobel Drive, Hercules, CA, USA). mRNA expression levels were analyzed by quantitative real-time PCR (qPCR) with a QuantStudio 3 Real-Time PCR system. Relative gene expression was calculated using the 2^−ΔΔCt^ method with GAPDH as the internal reference gene. All primers used for amplifying mouse gene sequences (detailed in Supplementary Table S4) were synthesized by Sangon Biotech (Shanghai, China).

### 2.11. Gut Microbiota Analysis

On day 14, intestinal contents were collected aseptically and snap-frozen for subsequent storage. Genomic DNA was extracted from these samples using the Mag-bind Soil DNA Kit (Omega Bio-Tek, Norcross, GA, USA), and its purity and concentration were subsequently determined. Amplification of the V3–V4 hypervariable region of the 16S rRNA gene was carried out with barcoded primers and high-fidelity DNA polymerase. After verification via 2% agarose gel electrophoresis, the obtained PCR products were purified, and their concentrations were measured with the Quant-iT PicoGreen dsDNA Assay Kit.

Fluorescence intensity was detected on a BioTek FLx800 microplate reader (BioTek Instruments, Inc., Winooski, VT, USA), and all samples were then pooled at equimolar concentrations. High-throughput sequencing libraries were prepared using the Illumina TruSeq Nano DNA LT Library Prep Kit.

Prior to sequencing, library quality and concentration were evaluated using the Agilent Bioanalyzer 2100 and Promega QuantiFluor system.

FASTQ files were generated from raw sequencing reads. Cutadapt was used to trim adapter sequences from paired-end data, while DADA2, implemented in QIIME2, was applied for quality filtering, denoising, read merging, and chimera removal. Each sample retained over 50,000 clean reads after quality control. QIIME2 was used to extract ASV representative sequences, which were taxonomically annotated against the SILVA 138 database using the classify-sklearn plugin. To ensure consistent sequencing depth across samples, the ASV feature table was normalized by rarefaction to the minimum read count among all samples. Alpha diversity was assessed using ACE, Chao1, and Shannon indices, and intergroup differences were compared via the Kruskal–Wallis test. Beta diversity was analyzed based on Bray–Curtis distance and visualized by PCoA and NMDS, with PERMANOVA used to evaluate group differences. Differential microbial taxa were identified using the Kruskal–Wallis test embedded in STAMP, with statistical significance set at *p* < 0.05. Further screening of microbial biomarkers was performed using Linear Discriminant Analysis Effect Size (LEfSe), with thresholds set at an LDA score > 2.5 and *p* < 0.05.

### 2.12. Proteomic Analysis

Mouse colonic tissues were homogenized in cold T-PER^TM^ reagent, and Bicinchoninic Acid Assay (BCA) assay was employed to measure protein concentration. For each sample, 150 μg of protein was subjected to reduction with dithiothreitol (DTT), alkylation with iodoacetamide (IAA), and tryptic digestion at 37 °C for 20 h. Peptides were acidified, desalted with C18 ZipTip, and analyzed by HPLC-MS/MS using an Orbitrap Exploris 240 and EASY-nLC 1200. Label-free quantification was done with Proteome Discoverer 2.5 against the UniProt mouse database, with a 1% false discovery rate (FDR) for peptide and protein identification. A label-free quantitative proteomics analysis was conducted with three biological replicates per group. Differentially expressed proteins (DEPs) were identified based on Benjamini–Hochberg adjusted *p*-value < 0.05 and fold change >1.2 or <0.83. For DEPs whose expression trends were reversed by IOM-H treatment, Gene Ontology functional annotation was performed using Blast2GO, and pathway enrichment analysis was carried out via the KEGG database.

### 2.13. Western Blot Analysis

After extraction, colon protein concentrations were determined using a BCA assay kit. Proteins were separated by Sodium Dodecyl Sulfate-PolyAcrylamide Gel Electrophoresis (SDS-PAGE) and transferred onto Polyvinylidene Fluoride (PVDF) membranes, which were then blocked with 5% skim milk in TBST for 3 h at room temperature. The membranes were incubated overnight at 4 °C with primary antibodies against PAD4, CitH3, and β-actin. After being washed three times with TBST, the membranes were probed with Horseradish Peroxidase (HRP)-conjugated secondary antibodies for 90 min at room temperature. Protein bands were visualized using a chemiluminescent substrate, and band densities were quantified with ImageJ software.

### 2.14. Dihydroethidium Staining

Dihydroethidium (DHE) staining was applied to assess ROS levels in colonic tissues. Sections prepared from freshly frozen colon tissues were stained with DHE under dark conditions at room temperature for 30 min. After incubation, sections were rinsed with PBS, mounted with fluorescence-preserving medium, and examined under an ECLIPSE Ti2-U inverted fluorescence microscope. Quantitative analysis of fluorescence intensity was conducted with ImageJ software.

### 2.15. Statistical Analysis

Quantitative results were analyzed using GraphPad Prism 9.5 and are presented as mean ± SD. Normality was assessed using the Shapiro–Wilk test. One-way ANOVA coupled with Tukey’s post hoc test was adopted for normally distributed data, whereas the Kruskal–Wallis test with Dunn’s post hoc comparison was applied for data lacking normal distribution. Statistical significance was defined as *p* < 0.05.

## 3. Results

### 3.1. Characterization of IOM

**Figure 1:**
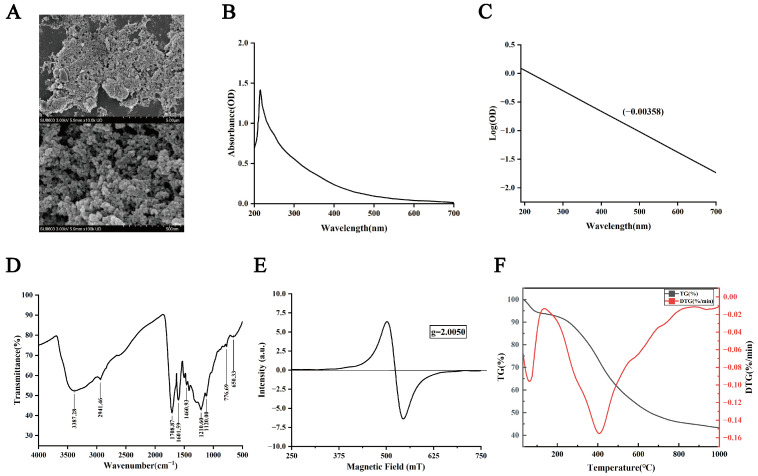
Characterization of IOM. (**A**) SEM image. (**B**) Full-range UV absorption spectrum. (**C**) Logarithmic plot of UV-visible absorption spectra. (**D**) FT-IR spectrum. (**E**) EPR spectrum. (**F**) TG-DTG curves.

The structural features of IOM were analyzed using various techniques, including SEM, UV, FT-IR, EPR, TG, HPLC, and elemental analysis. SEM revealed that IOM particles are primarily lumpy with rough surfaces, forming an irregular 3D structure with spherical particles (Figure 1A). The maximum absorption of IOM occurs at 215 nm and decreases linearly with increasing wavelength, a typical melanin trait, as shown in Figure 1B,C. The FT-IR spectrum revealed a strong, broad peak at 3387.28 cm^−1^, indicating -OH groups, and a peak at 2941.46 cm^−1^ for aliphatic C-H stretching. The 1708.86 cm^−1^ peak corresponded to C=O bending, while peaks at 1601 cm^−1^ and 1460 cm^−1^ suggested aromatic C=C stretching. Absorptions at 1210 cm^−1^ and 1120 cm^−1^ were linked to C-O stretching, possibly related to the aromatic ring. Peaks at 776 cm^−1^ and 650 cm^−1^ were due to out-of-plane aromatic C-H bending (Figure 1D). The EPR spectrum showed a strong single-line signal with g-factors near 2.0050, indicating free radicals characteristic of melanin (Figure 1E).

The thermal stability of IOM was analyzed by TG under nitrogen with a heating rate of 10 °C/min over the range of 25–1000 °C. Figure 1F shows the TG and DTG curves. IOM decomposed into two main stages: an initial 7% mass loss at 30–129 °C due to weakly bound water, and a major degradation at 229–918 °C, with a DTG peak around 400 °C indicating pyrolysis of its macromolecular structure. At 1000 °C, the IOM retained 43.25% of its mass, highlighting its excellent thermal stability due to its cross-linked aromatic structure. As shown in Supplementary Figure S1, the HPLC chromatogram of IOM displays a dominant peak at 13.41 min (accounting for 67% the total peak area), demonstrating high purity with few impurities. Elemental analysis revealed that IOM consisted of 60.965% C, 4.85% H, 0.105% N, and 0.095% S, with negligible N and S contents (Supplementary Table S5). The elemental profile of IOM aligns with that of allomelanin [31].

### 3.2. IOM Improves Clinical Manifestations of Colitis in UC Mice

**Figure 2:**
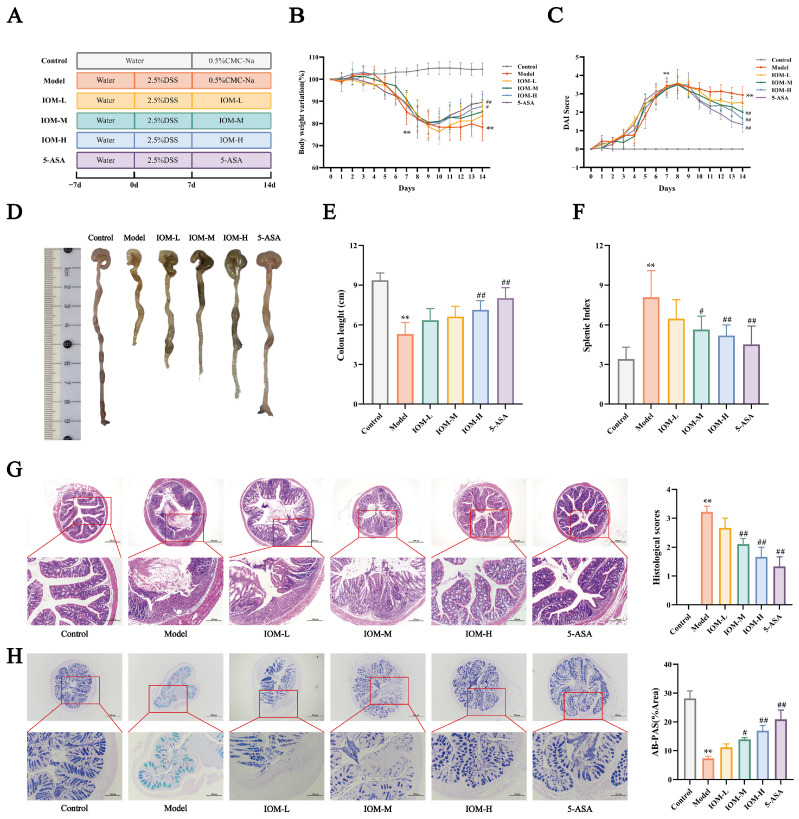
IOM protects against colitis induced by DSS in mice. (**A**) Schematic of the animal experimental protocol. (**B**) Body weight ratio over time. (**C**) DAI. (**D**) Macroscopic images of colonic tissues. (**E**) Colon index. (**F**) Splenic index. (**G**) Representative H&E staining and histopathological scores of colon tissues. (**H**) Representative AB-PAS staining and quantification of mucus area. Results are expressed as mean ± SD. *n* = 10 for panels (**B**,**C**), *n* = 6 for panels (**E**,**F**), *n* = 3 for panels (**G**,**H**). *p* < 0.05 (#) and *p* < 0.01 (**, ##) indicate significant differences compared with the Control and Model groups, respectively. Scale bars: 500 μm (top) and 200 μm (bottom) in (**G**,**H**).

The experimental design was illustrated in Figure 2A. Mice were administered 2.5% DSS for 7 days to establish a colitis model. Successful model induction was confirmed by decreased activity, lethargy, reduced food and water intake, progressive weight loss, diarrhea, hematochezia, and markedly elevated DAI scores compared with the Control group. Following treatment, mice in all IOM-treated groups exhibited marked improvements in food intake and daily behavioral activity relative to the DSS model group. The IOM-H and 5-ASA groups showed significantly greater body weights than the Model group (Figure 2B). Moreover, the IOM-M, IOM-H, and 5-ASA groups displayed markedly reduced DAI scores (Figure 2C) relative to the Model group. DSS-challenged mice also developed colon contraction and an increased spleen index. Treatment with IOM-H and 5-ASA significantly ameliorated colon shortening and reduced the spleen index compared with the Model group (Figure 2D–F). Collectively, these results demonstrate that IOM exerts a dose-dependent therapeutic effect on experimental colitis in mice.

### 3.3. IOM Attenuates Colon Damage in UC Mice

The HE staining and histopathological scoring results are shown in Figure 2G. The Control group showed intact colonic mucosa with regular structure and minimal inflammatory infiltration. In contrast, model mice displayed severe mucosal ulceration, crypt damage, and marked inflammatory cell infiltration. Treatment with 5-ASA and IOM-H effectively alleviated mucosal ulceration and inflammatory responses. Although mild inflammation was still observed in the IOM-M group, the severity of colonic mucosal damage was markedly reduced relative to the Model group. AB-PAS staining further supported these observations (Figure 2H). In model mice, colonic mucus was markedly reduced due to the loss and impaired secretory function of goblet cells. Conversely, treatment with 5-ASA, IOM-M, and IOM-H effectively preserved goblet cell numbers, restored mucus secretion, and increased colonic mucus deposition.

### 3.4. IOM Alleviates Inflammation and Oxidative Damage in UC Mice

**Figure 3:**
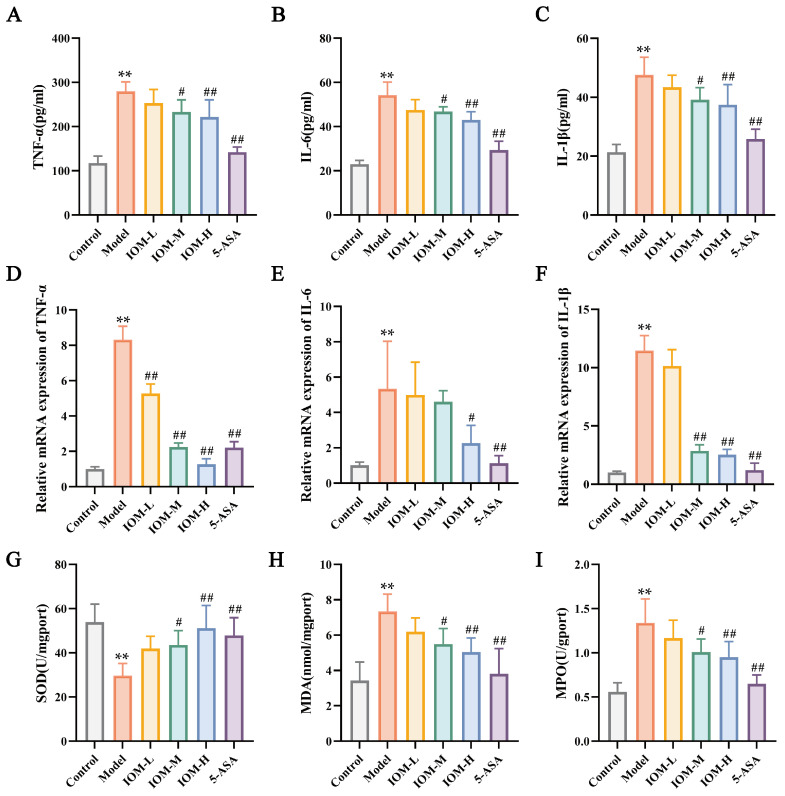
IOM alleviates inflammation and oxidative damage in UC mice. (**A**–**F**) Serum concentrations and colonic mRNA abundance of TNF-α, IL-6 and IL-1β. (**G**–**I**) Activities of SOD, MDA and MPO. Results are expressed as mean ± SD (*n* = 6). *p* < 0.05 (#) and *p* < 0.01 (**, ##) indicate significant differences compared with the Control and Model groups, respectively.

Figure 3A–F demonstrates that serum levels of TNF-α, IL-6, and IL-1β were markedly higher in the Model group than in the Control group. After administration of 5-ASA and IOM, the production of inflammatory factors was significantly reduced in all treated groups, with the IOM-H and 5-ASA groups showing the most obvious inhibitory effects. A critical aspect of UC progression is the dysregulated oxidative stress response [32]. Accordingly, the activities of SOD and MPO, as well as the content of MDA, were determined in colon tissues. As presented in Figure 3G, the Model group exhibited a notable decrease in SOD activity compared with the Control group. Following IOM treatment, SOD activity was significantly elevated, and this effect was enhanced with increasing dosage compared with the Model group. Conversely, the Model group showed a notable increase in MDA and MPO activity in colon tissues relative to the Control group, as indicated in Figure 3H–I. Treatment with IOM-M, IOM-H, or 5-ASA resulted in significantly reduced MDA and MPO levels compared with the Model group.

### 3.5. IOM Protects Against Intestinal Barrier Disruption in UC Mice

**Figure 4:**
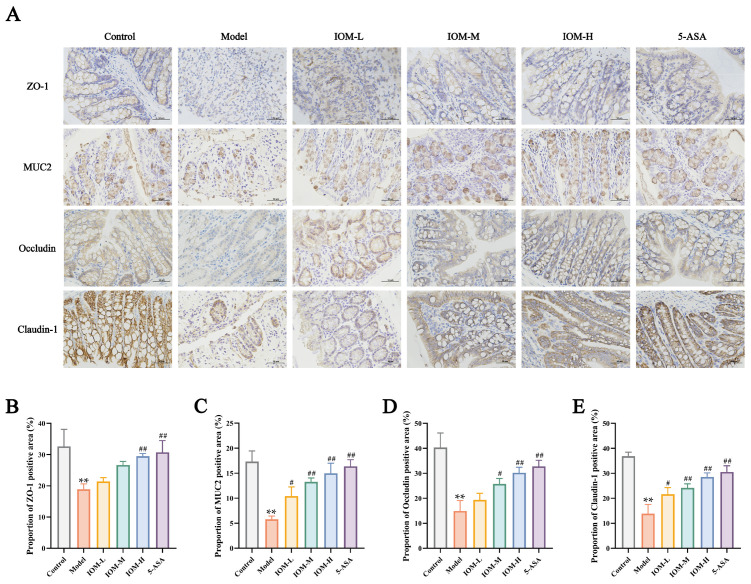
IOM maintains intestinal mucosal barrier function. (**A**–**E**) Immunohistochemical staining and quantification of TJ proteins and MUC2. Results are expressed as mean ± SD (*n* = 3). *p* < 0.05 (#) and *p* < 0.01 (**, ##) indicate significant differences compared with the Control and Model groups, respectively. Scale bar: 50 μm.

Colitis is characterized by impaired intestinal barrier function, where tight junction (TJ) proteins (including claudins, ZO-1, and occludins) and mucosal secretory proteins jointly regulate intestinal permeability [33]. By immunohistochemistry, we observed that the protein levels of ZO-1, Occludin, Claudin-1 and MUC2 were substantially down-regulated in the Model group relative to the Control group. IOM treatment significantly restored the expression of these proteins, suggesting that IOM could effectively ameliorate intestinal barrier dysfunction (Figure 4A–E).

### 3.6. IOM Modulates the Gut Microbiota in UC Mice

**Figure 5:**
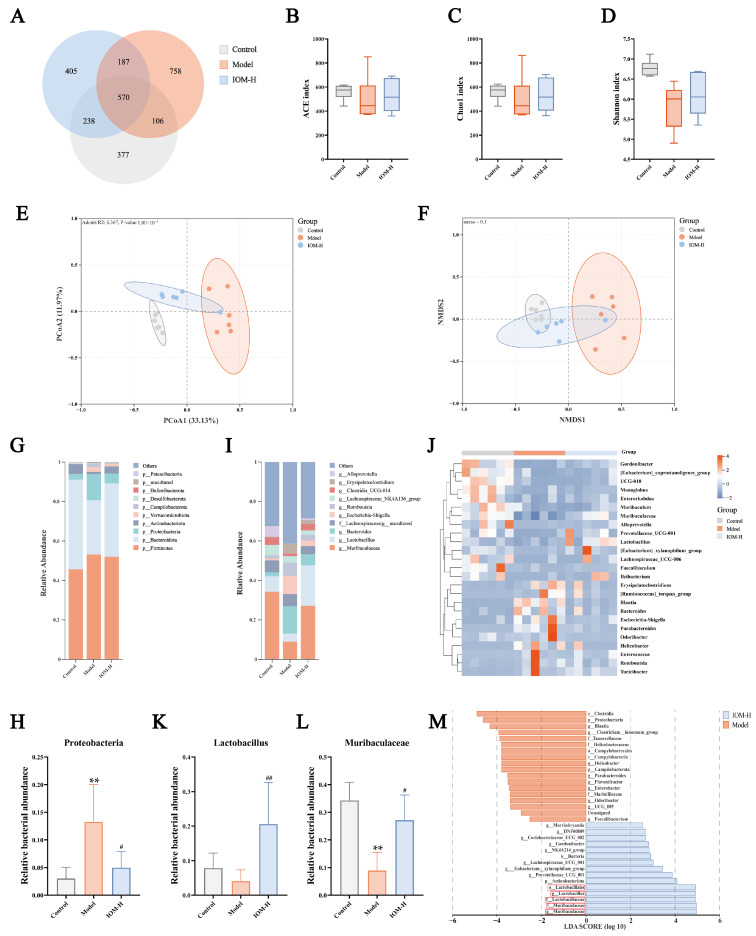
IOM alleviates gut microbial imbalance in DSS-induced colitis. (**A**) Venn diagram of ASVs. (**B**–**D**) ACE, Chao1 and Shannon indices. (**E**,**F**) PCoA and NMDS score plots. (**G**) Taxonomic profiles at the phylum level. (**H**) Relative abundance of Proteobacteria. (**I**) Taxonomic profiles at the genus level. (**J**) Heatmap of the top 25 genera with differential abundance. (**K**,**L**) Relative abundance of *Lactobacillus* and *Muribaculaceae*. (**M**) LDA score plot of distinct bacterial taxa in Model vs IOM-H groups (LDA score > 2.5). Results are expressed as mean ± SD (*n* = 6). *p* < 0.05 (#) and *p* < 0.01 (**, ##) indicate significant differences compared with the Control and Model groups, respectively.

Intestinal contents of control, UC model and IOM-H groups were subjected to 16S rRNA gene sequencing to explore IOM-mediated changes in gut microbiota in UC model mice. The Venn diagram (Figure 5A) displayed the number of common and specific Amplicon Sequence Variants (ASVs) among the Control, Model, and IOM-H groups. We further analyzed α-diversity indices to assess the complexity and composition of the gut microbiota. ACE, Chao1, and Shannon indices were significantly decreased in the Model group compared with the Control group. In contrast, IOM-H administration significantly elevated these indices and partially restored gut microbial α-diversity relative to the Model group (Figure 5B–D). Subsequent β-diversity analysis based on Principal Coordinates Analysis (PCoA) and Non-metric Multidimensional Scaling (NMDS) revealed distinct separation of gut microbial communities among the Control, Model, and IOM-H groups. As shown in Figure 5E,F, samples from the Control and IOM-H groups were closely clustered and exhibited higher similarity in microbial structure. In comparison, the Model group exhibited an obvious separation from these two groups. These results indicate that IOM treatment effectively ameliorated DSS-induced gut microbial dysbiosis and improved microbial diversity in UC mice.

Next, we analyzed the changes in gut microbial composition and relative abundance induced by IOM to better characterize the overall bacterial community structure among the three groups. The phylum-level microbial taxa present in each group varied in proportion but were largely consistent across all groups (Figure 5G), including Firmicutes, Bacteroidetes, Proteobacteria, Verrucomicrobia, and Actinobacteriota. Notably, the proportion of Proteobacteria in the Model group increased to 13.24%, whereas IOM treatment resulted in a reduction in Proteobacteria abundance to 4.96% (Figure 5H).

In the cecal microbiota of mice, the genera *Muribaculaceae*, *Lactobacillus*, *Bacteroides*, and *Escherichia-Shigella* were identified as the most prevalent (Figure 5I). As shown in Figure 5J, a heatmap was generated to show changes in microbial abundance among groups. The Model group exhibited increased abundances of *Escherichia-Shigella*, *Romboutsia*, *Bacteroides*, *Erysipelatoclostridium*, and *Turicibacter*, but reduced levels of *Muribaculaceae* and *Lactobacillus*. Such changes were prominently reversed by IOM treatment, as the levels of *Muribaculaceae* and *Lactobacillus* were elevated to values considerably higher than those observed in the Model group (Figure 5K,L). Collectively, these results demonstrate that IOM effectively alleviated gut microbial dysbiosis triggered by DSS, thereby driving the microflora toward a more balanced ecological status.

To further characterize these discrepancies, LEfSe was applied to identify the dominant bacterial taxa responsible for separating the Model and IOM-H groups. Taxa with an LDA (Latent Dirichlet Allocation) score above 2.5 were considered significant. Our results showed that DSS induced gut microbial dysbiosis, characterized by elevated levels of the phylum Proteobacteria. In contrast, the IOM-H group exhibited significant enrichment of Lactobacillales (order), Lactobacillaceae (family), *Lactobacillus* (genus), as well as *Muribaculaceae* at both family and genus levels. These specific taxa may contribute to the ameliorative effect of IOM-H on colitis (Figure 5M).

### 3.7. IOM Regulates the Proteomics Profile in UC Mice

**Figure 6:**
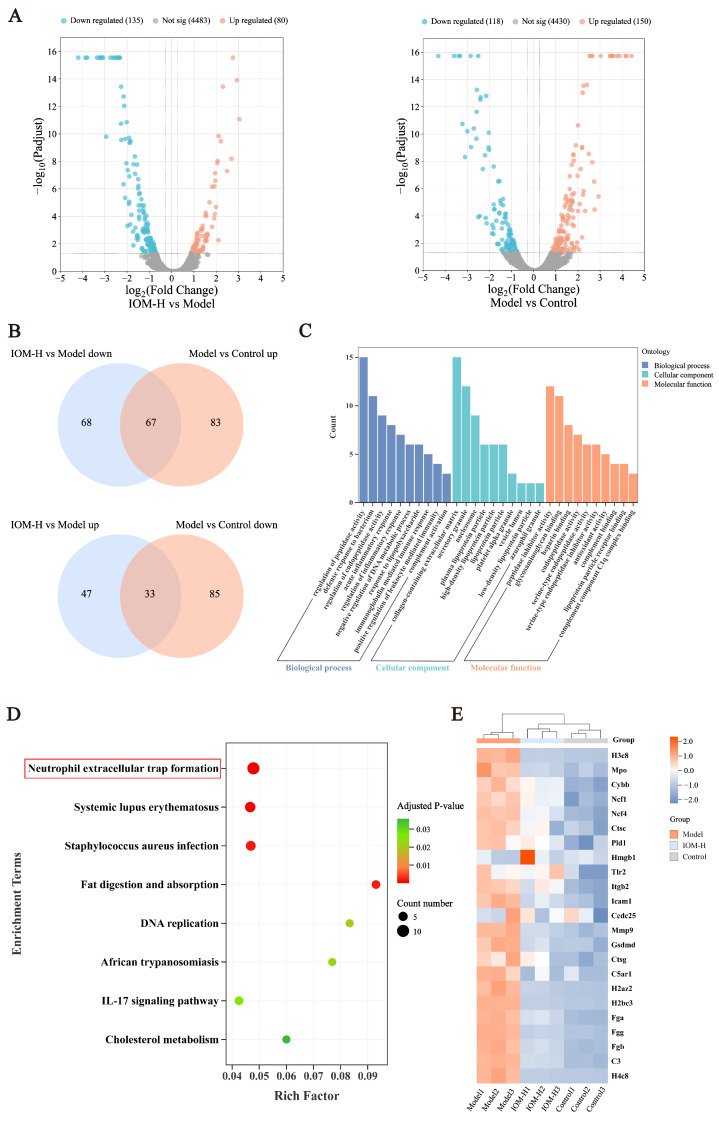
IOM modulates the proteomic profile of DSS-induced colitis mice (*n* = 3). (**A**) Statistical analysis and volcano plots of DEPs (Model vs. Control, IOM-H vs. Model). (**B**) Venn diagram of overlapping DEPs. (**C**,**D**) Functional annotation and pathway analysis of the DEPs. (**E**) Heatmap of DEPs.

To elucidate the underlying molecular basis of IOM against DSS-triggered acute colitis, we performed proteomic profiling and screened for DEPs and their associated signaling pathways. The volcano plot illustrated the DEPs across the experimental groups (Figure 6A). Overall, there were 118 significantly downregulated and 150 upregulated proteins in the Model group compared with the Control group. When comparing the Model and IOM-H groups, 135 proteins were downregulated and 80 upregulated in the IOM-H group.

We next focused on proteins displaying opposing expression patterns: those enhanced in the Model group but repressed after IOM-H treatment, and vice versa. In total, IOM-H treatment significantly reversed the aberrant upregulation of 67 proteins and restored the expression of 33 proteins that were downregulated in the Model group (Figure 6B).

To investigate the biological functions of these selected reversed DEPs, GO (gene ontology) enrichment analysis was performed to characterize their functional roles in cellular components, molecular activities, and biological pathways. Among the significantly enriched terms within biological processes, DEPs were mainly engaged in metabolism, biological regulation, and immune and inflammatory responses. The cellular component analysis revealed a predominance of cell parts and macromolecular complexes. Molecular functions were primarily associated with binding and enzyme-related activities (Figure 6C). To further explore the protective mechanisms of IOM in UC, KEGG pathway enrichment analysis was performed on the selected reversed DEPs. Of these, neutrophil extracellular trap (NET) formation was the most significantly enriched pathway (Figure 6D). Figure 6E presents a heatmap that delineates the expression patterns of key DEPs across the Control, Model, and IOM-H groups, specifically highlighting Mpo, Mmp9, and H3c8, the latter being a pivotal member of the histone H3 family. These proteins are known markers of neutrophil activation and inflammation, supporting that IOM may attenuate neutrophilic pro-NETotic signaling in the colon.

### 3.8. IOM Reduces Neutrophil Infiltration and Neutrophil pro-NETotic Activation

**Figure 7:**
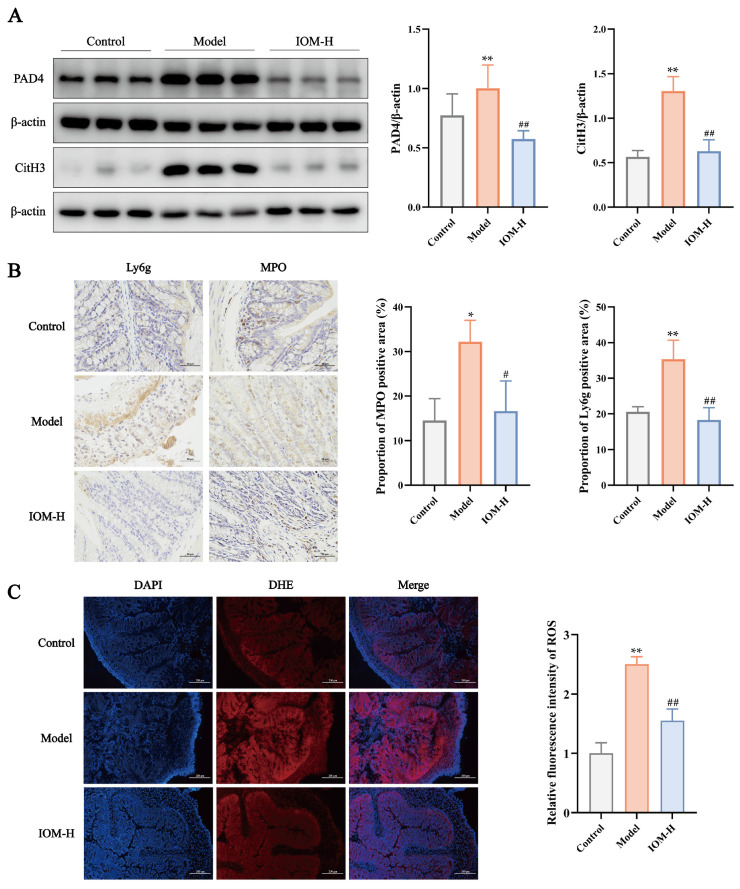
IOM mitigates DSS-induced colitis via suppressing neutrophil activation and NET formation in colon tissues. (**A**) Protein expression of CitH3 and PAD4. (**B**) IHC staining of Ly6G and MPO. (**C**) DHE staining for ROS levels. Results are expressed as mean ± SD. *n* = 6 for panel (**A**), *n* = 3 for panels (**B**,**C**). *p* < 0.05 (*, #) and *p* < 0.01 (**, ##) indicate significant differences compared with the Control and Model groups, respectively. Scale bar: 50 μm (**B**), 200 μm (**C**).

Proteomic analysis revealed that NET formation, a recently identified bactericidal mechanism of neutrophils, may be involved in the therapeutic efficacy of IOM. As important extracellular structures, NETs are composed of decondensed chromatin enveloped by cytoplasmic and granular proteins, including neutrophil elastase (NE), MPO, and CitH3, with the latter being mediated by PAD4 [34,35]. To validate the proteomic findings, we conducted investigations on NET-associated and neutrophil activation markers using Western blotting and immunohistochemical labeling techniques. Marked increases in CitH3 and PAD4 levels were observed in the Model group by Western blot. However, treatment with IOM resulted in a downregulation of these proteins (Figure 7A). Additionally, IHC analysis showed elevated expression of MPO and Ly6g in the Model group, which was markedly attenuated by IOM administration (Figure 7B). Furthermore, ROS may facilitate the production and release of NETs by inducing intracellular and extracellular oxidative stress [36]. IOM significantly attenuated the increased ROS levels in DSS-treated mice, as demonstrated by DHE staining (Figure 7C). The results demonstrated that IOM attenuated neutrophil activation and pro-NETotic signaling marker expression, thereby ameliorating DSS-induced colitis.

### 3.9. Correlation Analysis Between Key Gut Microbiota and NET-Related Proteins as Well as UC-Associated Indicators

**Figure 8:**
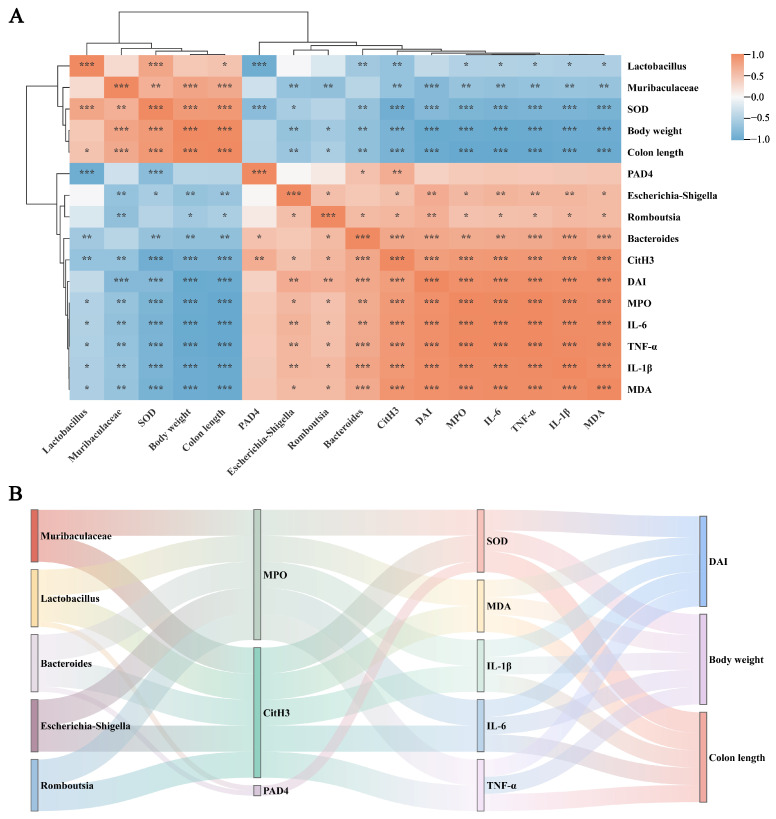
Associations among gut microbiota, NET-related proteins and colitis-related indicators. (**A**) Correlation heatmap analysis. (**B**) Predicted UC-related intestinal flora and NETs markers. *p* < 0.05 (*), *p* < 0.01 (**), *p* < 0.001 (***).

The correlation heatmap revealed that *Lactobacillus* and *Muribaculaceae* showed strong positive correlations with body weight, colon length and SOD levels, but strong negative correlations with DAI scores, MDA, MPO, CitH3, PAD4 and inflammatory cytokine levels. As shown in Figure 8A, *Bacteroides*, *Escherichia-Shigella* and *Romboutsia* exhibited a positive correlation with DAI scores, MDA, MPO, CitH3, PAD4 and inflammatory cytokine levels, but an inverse correlation with body weight, colon length and SOD levels. Furthermore, a Sankey diagram was constructed to depict the potential links among intestinal microbiota, neutrophil pro-NETotic activation and colitis-related indicators (Figure 8B), which mainly involved *Lactobacillus*, *Muribaculaceae*, *Bacteroides*, *Escherichia-Shigella* and *Romboutsia*. Collectively, these data suggest that the above bacteria are closely implicated in the alleviative effect of IOM on colitis.

## 4. Discussion

UC represents a chronic intestinal inflammatory condition whose global prevalence is rapidly rising, and is closely tied to intestinal dysbiosis, excessive inflammation, and impaired intestinal barrier function [15,27,37,38]. Current treatments are limited by side effects and high costs, increasing the need for safe alternatives [5,39]. IOM is a melanin isolated from *Inonotus obliquus*, a medicinal fungus long employed to alleviate gastrointestinal discomfort [8,10,13]. Nevertheless, the underlying mechanisms and therapeutic value of IOM in UC remain largely elusive. In the current work, we characterized the structure and thermal stability of IOM, providing a foundation for its biological activity. Our results demonstrated that IOM effectively protected mice from DSS-induced colitis, accompanied by gut microbiota remodeling, reduced neutrophil infiltration, and suppressed neutrophil pro-NETotic activation. These findings suggest that IOM may serve as a promising dietary bioactive ingredient for UC management.

The DSS-induced colitis model exhibits key inflammatory and phenotypic changes similar to human ulcerative colitis, such as weight loss and diarrhea. This model has been widely applied to study colonic injury and assess therapeutic candidates [40]. In this study, oral administration of IOM effectively ameliorated body weight loss, hematochezia, and increased DAI values, as well as preventing colon shortening. Moreover, IOM attenuated inflammatory cell infiltration and facilitated the recovery of intestinal mucosal integrity. These results support the potent efficacy of IOM against DSS-triggered colonic injury.

Evidence suggests that excessive inflammation and oxidative stress serve as critical drivers in the pathogenesis of UC [38,41]. Key proinflammatory mediators such as IL-1β, TNF-α and IL-6, released by intestinal epithelial cells, trigger inflammatory cascades and tissue damage during disease progression [42,43]. Levels of the oxidative stress indicators SOD, MDA, and MPO also correlate closely with disease severity [44]. In this study, IOM treatment significantly reduced proinflammatory cytokines and oxidative stress in colitic mice, indicating that IOM alleviates UC by suppressing inflammation and oxidative stress.

A healthy intestinal barrier is essential for intestinal homeostasis, in which tight junction proteins and mucus are critical components [45]. Impaired barrier function increases intestinal permeability, leading to bacterial invasion and sustained inflammation, thereby exacerbating UC [27,46]. Here, we observed that IOM administration elevated the levels of pivotal tight junction proteins and MUC2 in DSS-challenged mice, indicating that IOM maintains intestinal barrier integrity to ameliorate colonic damage.

The gut microbiota plays an indispensable role in maintaining intestinal homeostasis, and its dysbiosis is tightly linked to the onset and progression of UC [21,47]. In our sequencing analysis, DSS administration caused severe gut microbial dysbiosis, characterized by elevated pathogenic bacteria and reduced beneficial microbes. IOM treatment effectively restored gut microbial composition and notably decreased the abundance of Proteobacteria, a well-known indicator of intestinal inflammation. In addition, IOM treatment reduced pro-inflammatory genera, including *Escherichia-Shigella* and *Romboutsia*, while increasing beneficial taxa such as *Muribaculaceae* and *Lactobacillus*. These beneficial genera alleviate UC via multiple anti-inflammatory pathways [48,49]. Of note, *Muribaculaceae* helps maintain microbial homeostasis and regulates immune and inflammatory responses [50,51], while *Lactobacillus* exerts anti-inflammatory effects by modulating host immunity and gut microbial balance [52,53]. Thus, the modulation of gut microbiota by IOM may be closely linked to its beneficial effects on alleviating colonic inflammation and injury in DSS-induced colitis.

In addition to gut microbiota dysbiosis, abnormal neutrophil activation is another critical contributor to the progression of DSS-induced colitis [54]. As key innate immune cells, neutrophils undergo pro-NETotic activation upon excessive stimulation, which in turn exacerbates intestinal inflammation [55]. Previous proteomic studies have revealed elevated expression of NET-related marker proteins, including NE, MPO and MMP-9, in colonic tissues of UC patients [56]. In support of these observations, Dinallo et al. further demonstrated increased levels of MPO, NE, PAD4 and CitH3 in inflamed colonic tissues of UC patients [57]. Our label-free proteomic analysis further identified the neutrophil pro-NETotic signaling pathway as a key pathway underlying the beneficial action of IOM in DSS-provoked colonic injury. Moreover, our proteomic data identified several key DEPs associated with neutrophil pro-NETotic regulation, including Mpo, Mmp9 and H3c8, a core isoform of histone H3. During UC progression, Ly6G^+^ neutrophils are the major cellular source of pro-NETotic molecular mediators in inflamed colonic tissue, and PAD4 critically drives neutrophil pro-NETotic priming [35,58,59]. Upregulation of PAD4 induces histone citrullination and chromatin decondensation. These are hallmark molecular events of neutrophil pro-NETotic activation and inflammatory amplification, which in turn aggravate intestinal inflammation and disrupt the intestinal mucosal barrier [59]. Our study demonstrated that IOM treatment markedly reduced Ly6G^+^ neutrophil infiltration and decreased the expression of CitH3, MPO and PAD4 in colon tissues from DSS-treated mice.

Oxidative stress is closely linked to both inflammatory injury and pro-NETotic activation [60,61]. Consistent with these observations, our previous data demonstrated that IOM treatment normalized SOD activity and reduced MDA and MPO levels in DSS-stimulated colon tissues, indicating that IOM effectively alleviated oxidative stress. Since ROS generation is a critical trigger of NETosis, our data further confirmed that IOM significantly reduced DSS-induced ROS accumulation. Collectively, these results indicate that IOM suppresses neutrophil activation and pro-NETotic signaling, at least in part by attenuating oxidative stress and ROS production, thereby ameliorating DSS-induced colonic injury and colitis.

Herein, we performed correlation analysis to investigate the relationships among gut microbiota, neutrophil pro-NETotic signaling and UC-related indices in DSS-induced colitis mice following IOM administration. Emerging evidence indicates that gut microbiota directly modulates neutrophil pro-NETotic activation, thereby affecting intestinal inflammation [62]. Pathogenic bacteria can trigger excessive neutrophil pro-NETotic priming, whereas probiotics often exert protective effects by suppressing pathological pro-NETotic molecular activation [63,64]. Notably, *Lactobacillus* have been reported to inhibit ROS production and PAD4 activity, thus attenuating pro-NETotic molecular events and alleviating colitis [65,66].

Consistent with previous studies, our results revealed that beneficial bacteria, including *Lactobacillus* and *Muribaculaceae* were negatively correlated with NET-related markers, while inflammation-associated taxa, such as *Escherichia-Shigella* and *Romboutsia* showed positive correlations with neutrophil pro-NETotic activation. These findings imply that IOM may alleviate colitis alongside the restoration of gut microbiota and suppression of aberrant pro-NETotic signaling. Collectively, these findings highlight the crosstalk between gut dysbiosis and pathological pro-NETotic activation in UC, identifying both as important action targets of IOM.

However, several limitations of the current work should be acknowledged. First, the chemical structure of IOM has not been fully elucidated. Further studies incorporating more NET biomarkers and immunofluorescence co-localization assays are required to clarify the regulatory effect of IOM on neutrophil pro-NETotic activation. In addition, the extent to which the protective effect of IOM depends on the gut microbiota remains to be determined, and the crosstalk between the gut microbiota and the pro-NETotic signaling pathway deserves further exploration. The bioavailability, stability in food formulations, and human-equivalent dosage of IOM remain unclarified. Although the crude extract of *Inonotus obliquus* has been verified to be practically non-toxic in rodent acute toxicity tests [67], further systematic safety and pharmacokinetic investigations are still needed to support its translational application.

## 5. Conclusions

This study demonstrated that IOM mitigated DSS-induced UC in mice by alleviating colitis symptoms, enhancing intestinal barrier integrity, and reducing colonic inflammation and oxidative stress in a dose-dependent manner. The therapeutic effects of IOM occur alongside the modulation of gut microbiota homeostasis and the suppression of neutrophil pro-NETotic signaling. This process increases the relative abundance of beneficial taxa such as *Lactobacillus* and *Muribaculaceae*, while reducing inflammation-associated taxa, including *Bacteroides*, *Escherichia-Shigella*, and *Romboutsia*. Moreover, the relative abundances of these bacterial taxa were significantly correlated with UC progression and neutrophil pro-NETotic activation. These findings highlight the potential of IOM as a natural, safe, and nutritionally active functional food ingredient for the management of UC. Further research is warranted to explore its potential application in the development of functional foods to support UC management.

## Data Availability

The original contributions presented in this study are included in the article/Supplementary Material. Further inquiries can be directed to the corresponding author.

## Supplementary Materials

The following supporting information can be downloaded at: https://www.mdpi.com/article/10.3390/nu18111733/s1, Table S1: Chromatographic conditions for HPLC analysis; Table S2: Disease activity index (DAI) scoring criteria; Table S3: Pathological scores; Table S4: Primer sequence of qRT-PCR gene; Table S5: Elemental composition of melanin from IOM; Figure S1. HPLC chromatogram.

## Abbreviations

The following abbreviations are used in this manuscript:

UC	Ulcerative Colitis
DSS	Dextran Sulfate Sodium
IOM	*Inonotus obliquus* crude melanin
SEM	Scanning Electron Microscope
FTIR	Fourier transform infrared
EPR	Electron Paramagnetic Resonance
TGA	Thermogravimetric analysis
HPLC	High Performance Liquid Chromatography
DAI	Disease Activity Index
H&E	Haematoxylin and eosin
AB-PAS	Alcian Blue-Periodic Acid Schiff
TNF-α	Tumor necrosis factor-α
TJ	Tight Junction
MPO	Myeloperoxidase
ZO-1	zonula occludens-1
MUC2	mucin 2
CitH3	Citrullinated Histone H3
Ly6g	Lymphocyte antigen 6 complex, locus G
PAD4	Peptidylarginine deiminase 4
PCoA	Principal Co-ordinates Analysis
NMDS	Non-metric Multidimensional Scaling
LEFSE	Linear Discriminant Analysis Effect Size
LDA	Latent Dirichlet Allocation
