# Supplementary material for: Inonotus obliquus Crude Melanin Ameliorates DSS-Induced Colitis with Modulation of Gut Microbiota and Neutrophil pro-NETotic Activation
Source: Nutrients. 2026 May 28;18(11):1733. doi: 10.3390/nu18111733 (PMC13257849; doi:10.3390/nu18111733)
Supplement: Supplementary file 1 [file nutrients-18-01733-s001.zip › nutrients-4271784-supplementary.pdf]

Supplementary Information

Table S1. Chromatographic conditions for HPLC analysis.

Parameter	HPLC conditions
Column	Diamonsil C18 column (5 μm, 4.6 × 250 mm)
Column temperature	35 °C
Mobile phase A	0.1% formic acid in water
Mobile phase B	Acetonitrile
Gradient program	0–3 min, 90% A<br>3–48 min, 90%–0% A<br>48–53 min, 0%–100% B<br>53–60 min, 100%–90% B
Flow rate	1.0 mL/min
Injection volume	10 μL

Table S2. Disease activity index (DAI) scoring criteria.

Scores	Weight loss (%)	Stool consistency	Fecal occult blood test
0	<1	Normal	Negative
1	1~5	Loose	Light blue
2	5~10	Soft but formed	Blue
3	10~15	Mucoid stool	Dark blue
4	>20	Watery diarrhea	Gross bleed

Table S3. Pathological scores.

Score	Inflammation severity	Inflammation extent	Crypt damage
0	None	None	None
1	Mild	Mucous layer	1/3
2	Moderate	Submucosa	2/3
3	Severe	Muscularis and serosa	100 %
4	—	—	100 % + epithelium loss

Table S4. Primer sequence of qRT-PCR gene.

Gene	Primer	Sequence
GAPDH	Forward Primer	5′-AGGAGCGAGACCCCACTAACATCAA-3′
	Reverse Primer	5′-ATGGGGGCATCGGCAGAAGGGGCGG-3′
TNF-α	Forward Primer	5′-AGCAGAAGCTCCCTCAGCGA-3′
	Reverse Primer	5′-TCACATTTCTTTTCCAAGCGATCTT-3′
IL-6	Forward Primer	5′-CTCCCAACAGACCTGTCTATAC-3′
	Reverse Primer	5′-CCATTGCACAACTCTTTTCTCA-3′
IL-1β	Forward Primer	5′-GCCAGTGAAATGATGGCTTAGG-3′
	Reverse Primer	5′-AGGAGCACTTCATCTGTTTAGG-3′

Table S5. Elemental composition of melanin from IOM.

Elements (%)	IOM
C	60.965
H	4.850
N	0.105
S	0.095
O	33.985

O%=100%−C%−H%−N%−S%

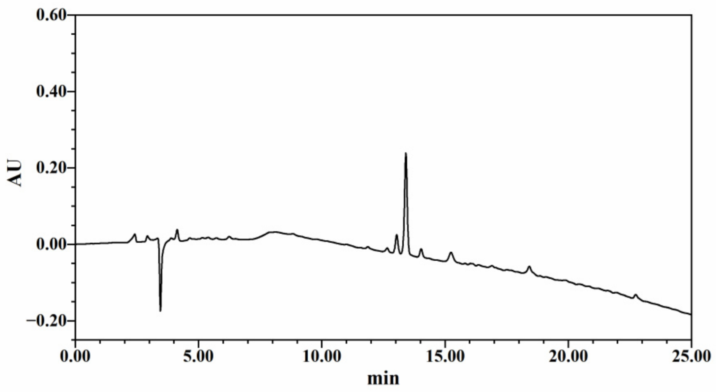

Figure S1. HPLC chromatogram.
